# Quantitative Analysis of Colonic Perfusion Using ICG Fluorescence Angiography and Its Consequences for Anastomotic Healing in a Rat Model

**DOI:** 10.3390/cancers14164024

**Published:** 2022-08-20

**Authors:** Toshiaki Wada, Kenji Kawada, Keita Hanada, Kazutaka Obama

**Affiliations:** 1 Department of Gastrointestinal Surgery, Graduate School of Medicine, Kyoto University, Kyoto 606-8507, Japan; 2 Department of Surgery, Faculty of Medicine, Kindai University, Osaka 589-8511, Japan; 3 Department of Surgery, Rakuwakai Otowa Hospital, Kyoto 607-8062, Japan

**Keywords:** rat model, colonic blood flow, ICG, fluorescence angiography, anastomotic leakage

## Abstract

**Simple Summary:**

Although indocyanine green (ICG) fluorescence angiography has been widely used to prevent anastomotic leakage (AL) in recent years, its usefulness remains unclear. The purpose of this study was to investigate whether quantitative analysis of colonic perfusion using ICG angiography could predict AL in a rat AL model. In the quantitative assessment, the following five parameters were calculated: Fmax (fluorescence difference between maximum and baseline), Tmax (time from onset to maximum), T_1/2_ (time from onset to half of maximum), Slope (Fmax/Tmax), and TR (T_1/2_/Tmax). Using a rat AL model, we found that there was a significant difference between the AL group and non-AL group in terms of Fmax, Tmax, T_1/2_, and Slope. In particular, Slope could be useful to predict AL.

**Abstract:**

Forty-three rats were randomly assigned to the following four groups: non-ischemic group (Control Group), 1 cm-long ischemic group (Group 1), 2 cm-long ischemic group (Group 2), and 3 cm-long ischemic group (Group 3). The rates of AL were 0% (0/10) in the Control Group, 22.2% (2/9) in Group 1, 25% (2/8) in Group 2, and 50% (4/8) in Group 3. The bursting pressure of the Control Group was significantly higher than that of the other groups (*p* < 0.01). Regarding the pathological findings, the granulation thickness and the number of blood vessels at the anastomosed site were significantly higher in the Control Group than in Group 3 (*p* < 0.05). Receiver operating characteristics analysis revealed that Slope was the most significant predictor of AL, with an area under the curve of 0.861. When the cutoff value of Slope was 0.4, the sensitivity and specificity for the prediction of AL were 75% and 81.4%, respectively. Quantitative analysis of ICG fluorescence angiography could predict AL in a rat model.

## 1. Introduction

Anastomotic leakage (AL) is the most serious complication of colorectal surgery. Rectal cancer patients suffering from AL have increased costs, an extended length of hospital stay, and an increased risk of local recurrence, although it is possible to cure AL. Despite advances in postoperative care and surgical techniques, the AL rate after rectal surgery remains at approximately 10% worldwide [1,2]. Although multifactorial factors are associated with the formation of AL, a general consensus in experimental and clinical studies indicates that a sufficient blood supply can promote anastomotic healing [3,4,5]. Therefore, an accurate evaluation of the intestinal blood perfusion is critical to avoid AL. Several methods have been proposed to measure the intestinal perfusion intraoperatively. Tests utilizing tissue oxygen and Doppler technology are not routinely used in clinical practice because of the expensive equipment and lack of reproducibility. Indocyanine green (ICG) is a water-soluble cyanine dye that fluoresces in the near-infrared region (790–805 nm) and has an absorption peak at 800–810 nm in blood. Recently, ICG fluorescence imaging has been widely used to investigate real-time intestinal perfusion during laparoscopic or robotic colorectal surgery. Several studies have reported that ICG fluorescence imaging can reduce the AL rate in the colorectal field [6,7,8,9,10,11,12]. However, in most of these clinical reports, colonic blood flow at the anastomosed site was subjectively evaluated with the surgeon’s naked eyes. A few animal experiments [3,4,13,14] and clinical studies [15,16,17,18] quantify ICG fluorescence intensity using luminance analysis software, such as ROIs, VR-RENDER, and Tracker4.97. Luminance analysis software can provide a quantitative and reproducible fluorescence value and a time-dependent change in the luminance value. In this study, a luminance analysis software, ROIs, was used to evaluate the parameters of colonic perfusion. The purpose of this study was to investigate whether quantitative analysis of colonic perfusion using ICG angiography could predict AL precisely in a rat AL model.

## 2. Materials and Methods

### 2.1. Animals and Ethical Considerations

**Figure 1:**
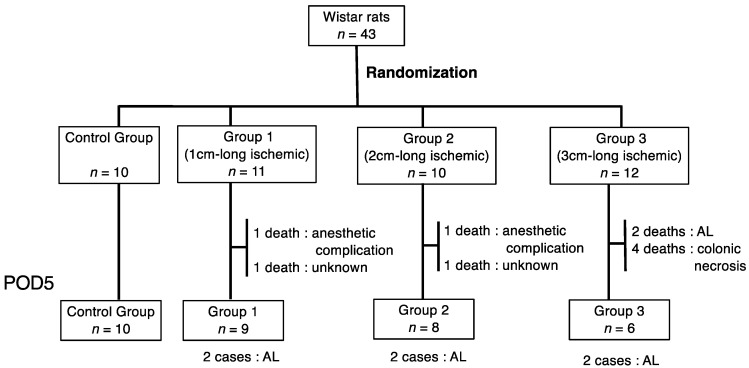
Study design and randomization of groups.

This study protocol was approved by the Animal Care and Use Committee of Kyoto University. All animal experiments were performed in accordance with the International Guiding Principles for Biomedical Research Involving Animals. Surgical procedures were carried out at the animal experimentation facility of Kyoto University. Female Wistar rats weighing 200–230 g (8 weeks old; Japan SLC, Shizuoka, Japan) were used. All of the animals were maintained under conditions of controlled humidity (50 ± 10%), temperature (24 ± 2 °C), and a 12 h light/dark cycle. The rats were fed with regular rat chow and kept under pathogen-free conditions with free access to water. Forty-three rats were randomly assigned to the following four groups: the non-ischemic group (Control Group), 1 cm-long ischemic group (Group 1), 2 cm-long ischemic group (Group 2), and 3 cm-long ischemic group (Group 3) (Figure 1).

### 2.2. Anesthesia and Surgical Procedures

**Figure 2:**
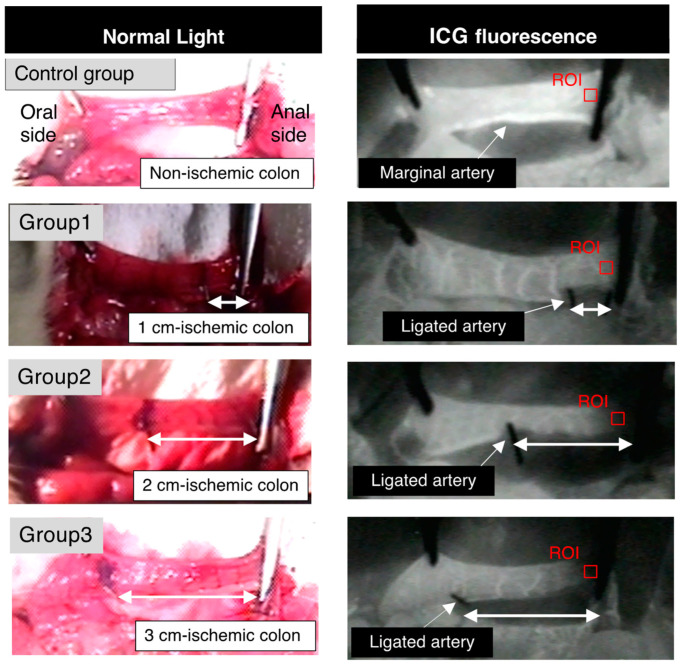
Non-ischemic colon group (Control Group), 1 cm-long ischemic colon group (Group 1), 2 cm-long ischemic colon group (Group 2), and 3 cm-long ischemic colon group (Group 3). ICG fluorescence was measured at the distal end of the pedicled segment (red square; region of interest).

All surgical procedures were performed with a surgical microscope under general anesthesia by inhalation of 2.5–3% isoflurane and an intraperitoneal injection of pentobarbital (64.8 mg/kg) after the rats had fasted for 12 h. The rats were placed on a heating pad, and a 3 cm midline laparotomy was conducted. We approached the distal colon and mesenterium. A marginal artery of the descending colon was ligated 0, 1, 2, or 3 cm away from the point that was 3 cm above the peritoneal reflection (Control Group, Group 1, Group 2, or Group 3, respectively). We created an ischemic descending colon model as previously reported by Posma et al. [3] (Figure 2). After ICG fluorescence imaging was recorded, the rectum was divided 3 cm above the peritoneal reflection. A single-layer, end-to-end anastomosis was performed using 12 stitch-interrupted sutures with 6-0 PDS. The rats were provided with water only on the day of surgery. The rats were allowed to begin feeding on postoperative day (POD) 1. The rats were sacrificed on POD 5. The anastomotic site was examined to determine whether anastomotic leakage or abdominal abscess was present. A 5 cm segment of the colon with an anastomosis was used for bursting pressure measurement and histopathological assessment.

### 2.3. ICG Fluorescence Angiography

**Figure 3:**
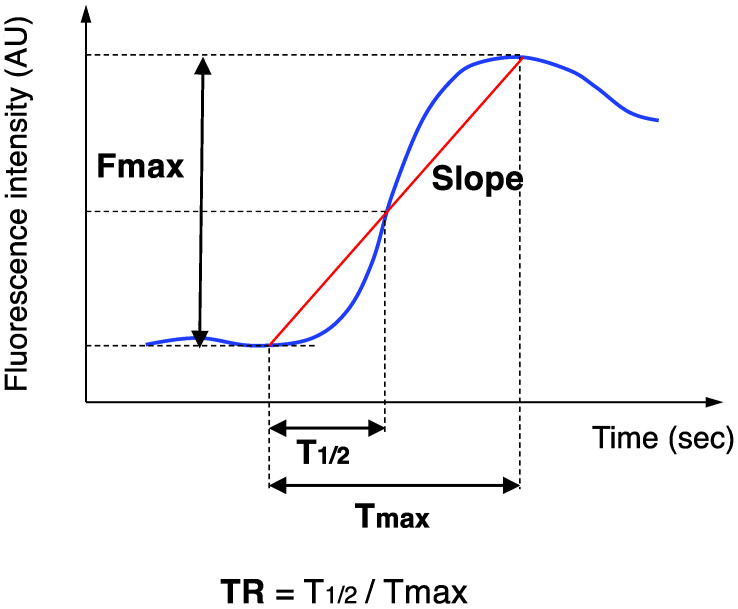
Time curve of ICG fluorescence intensity. Fmax, Tmax, T_1/2_, Slope, and TR were calculated.

Blood flow was measured intraoperatively using ICG fluorescence imaging. We used an NIR camera system (PDE-neo System; Hamamatsu Photonics K.K., Hamamatsu, Japan) and luminance analysis software (ROIs; Hamamatsu Photonics K.K.) for quantitative evaluation of the ICG fluorescence imaging. In a darkened room, the camera was fixed 15 cm away from the organ, and a bolus of ICG (0.2 mL of a 5 mg/mL solution) was injected into the penile vein after making an ischemic colon. Imaging data were continuously recorded for more than 5 min after ICG injection. For retrospective analysis, the stored movie videos were analyzed using the analysis software, ROIs. This luminance analysis software graphically displays the time-dependent change in the luminance, and calculates the following 5 parameters: Fmax (fluorescence difference between maximum and baseline), Tmax (time from onset to maximum), T_1/2_ (time from onset to half of maximum), Slope (Fmax/Tmax), and TR (T_1/2_ /Tmax) (Figure 3).

### 2.4. Bursting Pressure Measurements

On POD 5, a 5 cm segment of colon including the anastomosis and adherent organs was extracted. A 3 mm catheter was placed into the oral lumen. The oral side of the colon was occluded with 3-0 silk sutures, and the anal side of the colon was clamped using forceps. The catheter was connected to an infusion syringe and a manometer (HANDY MANOMETER PG-100B, COPAL ELECTRONICS, Tokyo, JAPAN). The pressure that caused anastomotic leakage was defined as the “bursting pressure”.

### 2.5. Histopathological Assessment

The anastomosed colon was extracted, fixed in a 4% formaldehyde solution, and embedded in paraffin. Sections (5 μm) were stained with hematoxylin and eosin (H&E), von Willebrand factor (vWF) reagent, and collagen type III. All assessments were performed by two investigators blinded to the experimental groups (TW and KH). The distance of granulation tissue at the anastomosis was measured at POD 5. The sections were stained with H&E, and viewed systemically at ×100 magnification. The granulation tissues in three sections (both sides and center) was measured, and the average of the three sections was calculated. Inflammatory cells (i.e., polymorphonuclear leukocyte cells, plasma cells, and lymphocytes), fibroblasts, and collagen deposition were scored at the anastomotic site using the modified 0-4 Ehrlich and Hunt numerical scale [19]. At ×400 magnification, the number of lumens in the newly formed vWF^+^ blood vessels was counted in each 1 mm^2^ field. The anastomotic sites of three fields (both ends and center) were counted, and the average was calculated.

### 2.6. Statistical Analysis

Values are expressed as means ± standard deviation (SD). Fisher’s exact test was used for comparison and analysis of categorical variables. The Mann–Whitney U test was used for continuous variables. All analyses were two-sided, and a *p*-value of <0.05 was considered to be statistically significant. Statistical analyses were conducted with JMP Pro software, version 11.0.0 (SAS Institute Inc., Cary, NC, USA).

## 3. Results

### 3.1. Anastomotic Leakage (AL)

A total of 43 rats were analyzed in this study: 10 rats in the non-ischemic group (Control Group), 11 rats in the 1 cm-long ischemic group (Group 1), 10 rats in the 2 cm-long ischemic group (Group 2), and 12 rats in the 3 cm-long ischemic group (Group 3). Two rats in Group 1 died; one on POD 1 due to an anesthetic complication after surgery, and the other on POD 2 due to an unknown reason. Two rats in Group 2 died; one on POD 1 due to an anesthetic complication after surgery, and the other on POD 2 due to an unknown cause. Six rats in Group 3 died up to POD 5; two on POD 2/3 due to AL, and four on POD 2 due to colonic necrosis (Figure 1). Rats that died due to anesthetic complications or colonic necrosis were excluded from the statistical analysis. Finally, the rates of AL were 0% (0/10) in the Control Group, 22.2% (2/9) in Group 1, 25% (2/8) in Group 2, and 50% (4/8) in Group 3.

### 3.2. Colonic Blood Flow Measured by ICG Fluorescence Angiography

**Figure 4:**
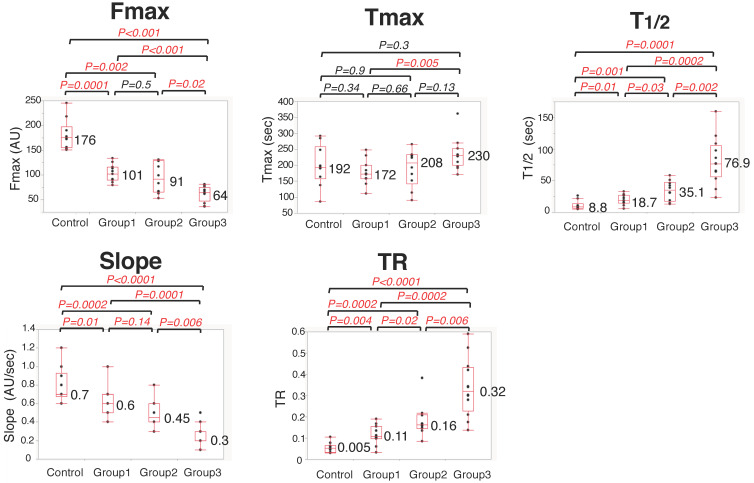
Comparison of five ICG fluorescence-related parameters between four groups.

Regarding colonic blood flow measured by ICG fluorescence, each parameter in the four groups was as follows (mean values: Control Group vs. Group 1 vs. Group 2 vs. Group 3): (1) Fmax (AU): 176 vs. 101 vs. 91 vs. 64; (2) Tmax (sec): 192 vs. 172 vs. 208 vs. 230; (3) T_1/2_ (sec): 8.8 vs. 18.7 vs. 35.1 vs. 76.9; (4) Slope (AU/sec): 0.7 vs. 0.6 vs. 0.45 vs. 0.3; and (5) TR: 0.005 vs. 0.11 vs. 0.16 vs. 0.32 (Figure 4). Regarding Fmax, T_1/2_, Slope, and TR, there was a significant difference between the Control Group and Group 3. Overall, as colonic ischemia became severe, Fmax and Slope tended to have low values, whereas T_1/2_ and TR tended to be extended.

### 3.3. Anastomotic Bursting Pressure

**Figure 5:**
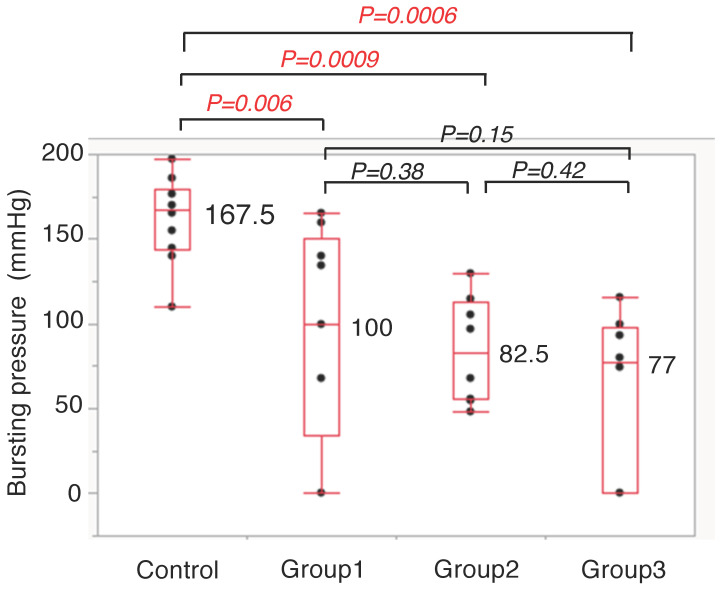
Bursting Pressure on POD 5.

The bursting pressure in the four groups was measured on POD 5 as follows (mean values: Control Group vs. Group 1 vs. Group 2 vs. Group 3): 167.5 vs. 100 vs. 82.5 vs. 77 mmHg (Figure 5). The bursting pressure of the Control Group was significantly higher than that of the other groups (*p <* 0.01). There were no significant differences among the other three groups (Group 1, Group 2, and Group 3).

### 3.4. Five ICG Fluorescence-Related Parameters between AL Group and Non-AL Group

**Figure 6:**
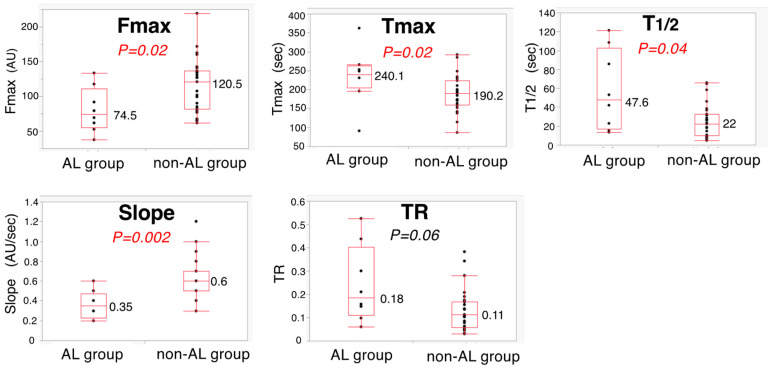
Comparison of five ICG fluorescence-related parameters between AL group and non-AL group.

**Figure 7:**
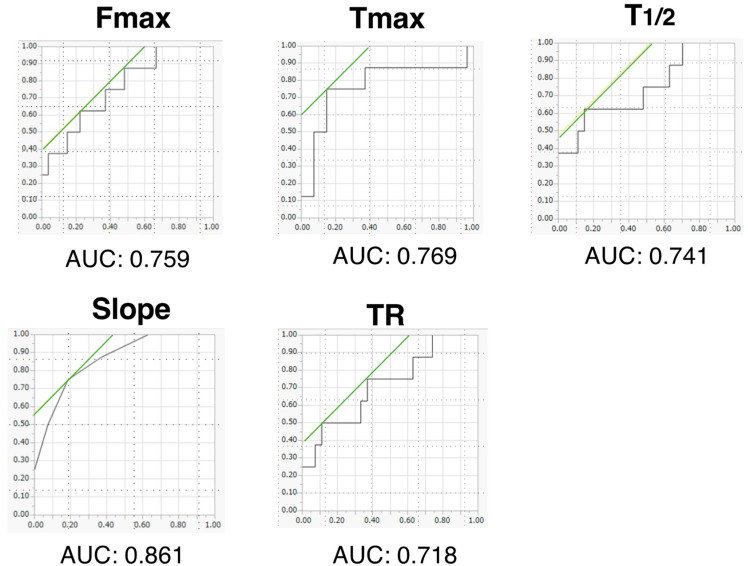
Prediction of AL by receiver operating characteristic (ROC) curve.

Regarding the relationship between AL and colonic blood flow, a significant difference was observed between the AL group (*n* = 8) and non-AL group (*n* = 27) in Fmax, Tmax, T_1/2_, and Slope: 74.5 vs. 120.5 (*p* = 0.02); 240.1 vs. 190.2 (*p* = 0.02); 47.6 vs. 22 (*p* = 0.04); and 0.6 vs. 0.35 (*p* = 0.002), respectively (Figure 6). Receiver operating characteristic analysis indicated that Slope was the most significant predictor of AL, with an area under the curve (AUC) of 0.861. Slope of the AL group was less than or equal to 0.4 (AU/sec) in six cases (6/8), whereas that of the non-AL group was in five cases (5/27): when the cutoff value of Slope was 0.4, the sensitivity and specificity for the prediction of AL were 75% and 81.4%, respectively (Figure 7).

**Figure 8:**
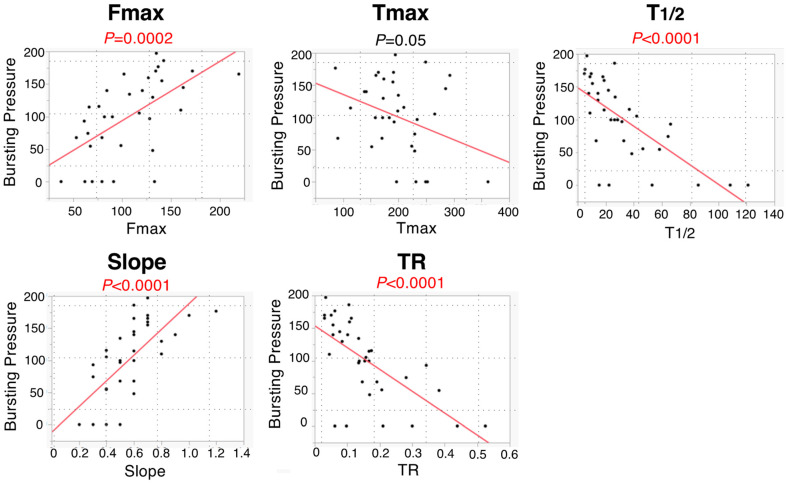
Relationship between five ICG fluorescence-related parameters and bursting pressure.

For continuous variables related to bursting pressure and colonic blood flow, a significant correlation was observed in Fmax, T_1/2_, Slope, and TR using Pearson’s correlation analysis (*p* = 0.0002, <0.0001, <0.0001, and <0.0001, respectively) (Figure 8).

### 3.5. Histopathological Evaluation

**Figure 9:**
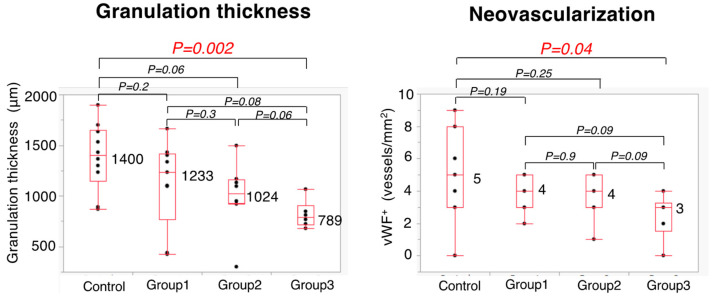
Histopathological assessment on POD 5.

**Figure 10:**
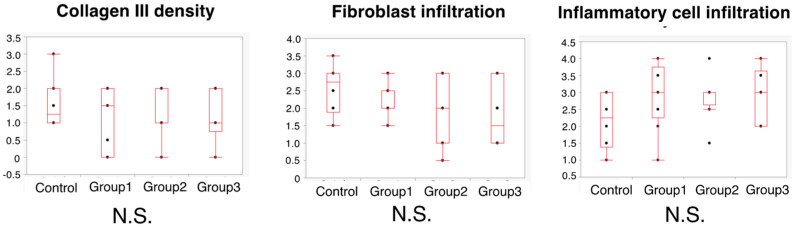
Histopathological assessment on POD 5 using Ehrlich and Hunt numerical scale. N.S.—not significant.

At the anastomotic site, the thickness of granulation was significantly higher in the Control Group than in Group 3 (1400 vs. 789 μm; *p* = 0.002) (Figure 9). In addition, the number of blood vessels was also significantly higher in the Control Group than in Group 3 (5 vs. 3 vessels; *p* = 0.04). There were no significant differences among the four groups in the Ehrlich and Hunt numerical scale scores for collagen III density, fibroblast infiltration, and inflammatory cell infiltration (Figure 10).

## 4. Discussion

Despite recent technical advances, AL is the most severe complication of colorectal surgery. Once AL occurs, patients experience increased morbidity and mortality, prolonged hospital stays, increased risk of permanent stoma, poor prognosis, and increased medical expenses. Thus, a method for predicting and reducing postoperative AL is required. Despite the multifactorial etiology of AL, sufficient intestinal perfusion is generally considered to be particularly important [20]. Clinical parameters, such as intestinal color, peristalsis, mesenteric pulse, and active bleeding of peripheral blood vessels have historically been assessed by experienced surgeons; however, their accuracy is low. More advanced evaluation methods for intestinal blood perfusion, such as laser Doppler fluxometry and tissue oxygen concentration measurements, have also been assessed. However, these techniques are not generally used in clinics because of their problems in objectivity and reproducibility.

ICG fluorescence imaging is a useful method owing to its easy access, quick use, good accuracy, low cost, and minimal invasiveness. In the colorectal surgical fields, ICG fluorescence imaging is rapidly spreading worldwide because the laparoscopic camera and robotic surgery Firefly technology (da Vinci Surgical System Xi) can visualize ICG fluorescence.

In a rat model, Posma et al. reported that ligation of one or three feeding arteries in the colon reduced the mean Slope to 59% or 26%, respectively, and that there was no correlation between bursting pressure and Slope [3]. Meanwhile, in our study, Slope decreased to 85.7% in Group 1, 64.3% in Group 2, and 42.9% in Group 3. Fmax, TR, Slope, and T_1/2_ were significantly associated with bursting pressure (Figure 8). In a pig model, Diana et al. reported that the lactate level of the 25% Slope area was significantly higher than that of the 75% Slope area, and that the histopathological inflammation score was higher in the 25% Slope area than in the 75% Slope area [21]. In a pig model, Nerup et al. reported that the blood flow in the stomach was significantly correlated to Fmax and Slope [22]. In a bowel strangulation model of a pig or rat, Matsui et al. reported that qualitative and quantitative metrics, including Fmax, could predict clinical outcomes, such as animal survival, poor histological grade, and tissue necrosis [23].

The present model was simple. As the colonic ischemia increased, the blood flow parameters gradually decreased, and the AL rates gradually increased. In this study, we evaluated intestinal perfusion of the ischemic colon using five parameters: Fmax, Tmax, T_1/2_, Slope, and TR. This study showed that Group 3 (3 cm-long ischemic colon group) resulted in a higher AL rate (50%). Regarding the relationship between ICG fluorescence parameters and AL, the Fmax, Tmax, T_1/2_, and Slope were significantly associated with AL. In particular, we propose that Slope could be the most accurate parameter for predicting colonic AL. Regarding the bursting pressure on POD 5, the median value of the Control Group was more than twice as high as that of Group 3.

Recently, two papers on randomized controlled trials (RCTs) using ICG fluorescence angiography were reported. Alekseev et al. reported that out of the total 380 patients who underwent sigmoid and rectal resection, a decrease in the AL rate was observed for low (4–8 cm) colorectal anastomoses (25.7% in the control group vs. 14.4% in the ICG group; *p* = 0.04), which suggests that ICG fluorescence angiography is associated with a reduction in the AL rate after low anterior resection [24]. Nardi et al. reported that in 252 patients undergoing laparoscopic left-sided colon and rectal resection, AL developed in 11 patients (9%) in the control group and 6 patients (5%) in the ICG group (*p*  =  not significant) [25]. Judging from these RCT results, it seems that ICG fluorescence angiography can be effective to prevent AL in rectal surgery. In the future, we expect that the blood flow parameters, such as Slope, can enable a more accurate evaluation of the colon and reduce the AL rate.

This study has some limitations. First, colonic blood flow is not the only cause of AL. Although several factors, including BMI, tumor size, level of anastomosis, intestinal perfusion, stapler firings, and intestinal microbes, are reported as possible causes of AL, the pathogenesis of AL is still unknown. Second, there were some individual differences in the anatomy of the blood vessels of each rat. Finally, the intestinal anastomosis was performed using the hand-sewn technique instead of mechanical anastomosis. However, it is important to note that quantitative analysis of ICG fluorescence angiography, especially Slope, has been shown to accurately predict AL in a rat model.

## 5. Conclusions

Fluorescence technology with ICG provides a real-time measurement of intestinal perfusion. Quantitative analysis of intestinal perfusion using ICG fluorescence angiography could predict AL in this rat model. Among the ICG-related parameters, Slope is particularly useful for predicting AL. Further studies are required to confirm whether these findings can apply to human clinical practice, which may result in a more precise decision to determine the transection line of the proximal colon in colorectal surgery.

## Data Availability

The data presented in this study are available from the corresponding author upon reasonable request.
